# Impact of rapid antigen detection testing on antibiotic prescription in acute pharyngitis in adults. FARINGOCAT STUDY: a multicentric randomized controlled trial

**DOI:** 10.1186/1471-2296-11-25

**Published:** 2010-03-23

**Authors:** Jordi Madurell, Montse Balagué, Mónica Gómez, Josep M Cots, Carl Llor

**Affiliations:** 1 Primary Healthcare Center Hostalric, Institut d'Assistència Sanitària de Girona, Breda-Hostalric, Spain; 2 Primary Healthcare Center, Centre Penitenciari d'Homes. Catalonian Institute of Health, Barcelona, Spain; 3 Primary Healthcare Center Doctor Josep Torner i Fors, Salut Maresme, Malgrat de Mar, Spain; 4 Primary Healthcare Center La Marina. University of Barcelona, Barcelona, Spain; 5 Primary Healthcare Center Jaume I. University Rovira i Virgili, Tarragona, Spain

## Abstract

**Background:**

Acute pharyngitis is one of the most frequent consultations to the general practitioner and in most of the cases an antibiotic is prescribed in primary care in Spain. Bacterial etiology, mainly by group A beta-hemolytic streptococcus (GABHS), accounts for 10-20% of all these infections in adults. The purpose of this study is to assess the impact of rapid antigen detection testing (RADT) to identify GABHS in acute pharyngitis on the utilization of antibiotics in primary care.

**Methods/design:**

Multicentric randomized controlled trial in which antibiotic prescription between two groups of patients with acute pharyngitis will be compared. The trial will include two arms, a control and an intervention group in which RADT will be performed. The primary outcome measure will be the proportion of inappropriate antibiotic prescription in each group. Two hundred seventy-six patients are required to detect a reduction in antibiotic prescription from 85% in the control group to 75% in the intervention group with a power of 90% and a level of significance of 5%. Secondary outcome measures will be specific antibiotic treatment, antibiotic resistance rates, secondary effects, days without working, medical visits during the first month and patient satisfaction.

**Discussion:**

The implementation of RADT would allow a more rational use of antibiotics and would prevent adverse effects of antibiotics, emergence of antibiotic resistance and the growth of inefficient health expenses.

**Trial registration:**

ISRCTN23587778

## Background

Acute pharyngitis is frequently seen in primary care for which uncertain etiology may result in inappropriate management. Acute pharyngitis includes rhinitis, the most common cause of visit in primary care in Spain [1]. Most pharyngotonsillitis are viral in origin while group A β-hemolytic *Streptococcus *(GABHS) is the most common bacterial cause of acute pharyngitis, accounting for approximately 15% to 30% of the cases in children and 5% to 15% of cases in adults [2]. GABHS is the only commonly occurring form of acute pharyngitis for which antibiotic therapy is indicated [3]. Nonetheless, a total of 78-98% of adults with acute pharingytis is treated with antibiotics, especially if sore throat is present [4,5]. Pharyngeal infection is also a frequent cause of absenteeism, with an estimated average of 6.5 days of sick leave per episode [2,6]. In streptococcal pharyngitis, antibiotic treatment shortens the transmission and dissemination of GABHS in the community, reduces the symptomatology compared to the non treated group by a mean of 16 hours and prevents suppurative complications. According to the Cochrane review, for each 100 patients treated with antibiotics compared to the placebo assigned group, one case less of rheumatic fever, two cases less of acute otitis media and 3 cases less of quinsy are produced [7,8].

Overuse of antibiotics can lead to side effects and emergence of antibiotic resistance [9]. The main reason for antibiotic overprescription in acute pharyngitis is the difficulty to obtain a rapid and correct etiological diagnosis. In addition, physicians often believe that patients expect an antibiotic and, in case of doubt, general practitioners are more prone to prescribe antibiotics. In Spain, rapid antigen detection tests (RADT) are seldom used in general practice and physicians still rely on the Centor criteria -fever, tonsillar exudates, tender vertical nodes, and/or absence of cough- for prescribing antibiotic therapy [10]. Sore throat culture is the gold standard for the diagnosis of streptococcal pharyngitis. However, the delay in obtaining results makes its performance useless in clinical practice.

Over the last years, low-cost and rapid immunological techniques targeting the detection of GABHS antigens have been developed for use in the clinical setting. Their high specificity (>95) make the probability of obtaining false positive results very low [11-13]. A recent paper published in Greece shows that the use of these tests can reduce the prescription of antibiotics in pharyngotonsillitis in children [14]. However, very few studies have been carried out to know the impact of RADT on the pattern of how general practitioners prescribe antibiotics for patients with pharyngotonsillitis in adults.

## Methods/Design

### Objectives

The main objective of this investigation is to assess the impact of the RADT in adults with acute pharyngitis on the percentage of antibiotics prescribed by general practitioners

The secondary objectives are:

a. To describe the antibiotics prescribed.

b. To describe GABHS resistance to the antibiotics prescribed.

c. To assess clinical recurrence within the first month.

### Design

Multicenter randomized clinical trial designed to compare antibiotic prescription in patients with acute pharyngitis in two groups of physicians, one with the use of RADT and the other without these tests.

### Setting

The trial will be conducted in Primary Health Care Centers in Catalonia (Spain) in which the clinical history records are accessed electronically.

### Participants

#### Study population

Eligible patients will be consecutively selected by general practitioners. All patients who agree to participate will be asked to provide written informed consent and an informative sheet of the study.

#### Inclusion criteria

1. Men and women aged 14 to 60 years.

2. More than one symptom of acute pharyngitis, such as fever, sore throat, tonsillar exudates, tender cervical nodes and/or absence of cough.

#### Exclusion criteria

1. Patients who do not consent to participate.

2. Patients younger than 14 or older than 60 years of age.

3. More than five episodes of pharyngitis over the last year.

4. Immunosuppressed condition, such as active neoplasm, acquired immunodeficiency syndrome or reception of chemotherapy, radiotherapy, corticosteroids and/or immunosuppressive therapy.

5. Heart valve disease.

6. Rheumatic fever.

7. Episode of pharyngitis treated with antibiotics in the previous fifteen days.

8. Pharyngitis of gonococcal etiology.

9. Tonsillectomy.

### Intervention

Participating primary health care centers will be randomized to the intervention or to the control arm of the study. Physicians allocated to the intervention group will use the RADT and those assigned to the control group will make the diagnosis of streptococcal pharyngitis with only clinical criteria. Treatment will be decided upon by the general practitioners in both groups, including antithermic drugs, non-steroidal anti-inflammatory drugs, corticosteroids, antibiotic therapy or none.

Samples will be taken by the family physicians who will be previously trained to perform the technique correctly with vigorous rotation of the tonsils and the posterior pharynx without touching the tongue, teeth or gums. RADT will be undertaken with all the samples with the OSOM StrepA (Genzyme) following the manufacturer's instructions. The other sample will be sent to the Department of Microbiology of the two hospitals of the area with AMIES (Copan Innovation, Italy) as medium. Samples will be seeded on a plate of blood agar and will be incubated at 37°C in an atmosphere of CO_2 _at 5% during 48 hours. A culture will be considered positive for GABHS with a growth of any number of β-hemolytic colonies, Gram staining with streptococcal morphology and a catalase negative test with posterior identification with an automated panel for WIDER Gram positive cocci (Soria Melguizo). Results will be confirmed with posterior serogrouping with the *Streptococcal Grouping Kit *(Oxford, UK). The culture will be considered negative after 48 hours of incubation with the absence of β-hemolytic colonies.

### Outcome measures

Primary outcome: proportion of inappropriate antibiotic prescription, use of antibiotic treatment, use of RADT, RADT result, and culture result.

#### Secondary outcomes

- Intervention arm only: RADT result.

- Both intervention and control groups:

**a**. Age, sex, clinical symptoms (fever, sore throat, tender cervical nodes, absence of cough), specific antibiotic treatment, culture result, antibiotic resistance, side effects, days without working, change of antibiotic, and medical visits within the first month.

**b**. Patient satisfaction. Patients will be required to score the degree of satisfaction, from 0 (no satisfaction at all) to 10 (very satisfied), for the following items: ease of obtaining a visit the day the patient wants, duration of the visit, doctor clinical ability, doctor kindness, doctor predisposition to listen, the way the problem is solved, the feeling of being in "good hands", and overall satisfaction with the doctor. Patients will be asked if they have been attended by their usual doctor or not and if they would recommend the attending doctor to a friend or relative. The open questions include: What is the most satisfying aspect of the visit? What is the worst aspect of the visit? Would you like to make any comment on anything else?

### Ascertainment of visits

#### Baseline data collection

The baseline questionnaire at the clinical practice will include age, sex, clinical symptoms, specific antibiotic treatment, use of RADT and RADT result.

#### Follow-up data collection

• 48-72 h data collection by means of a phone call that will include side effects, days without working, and change of antibiotic or not.

• Three-week data collection: The questionnaire will include the number of visits, if there has been any complication, culture result and antibiotic resistance to GABHS if the culture is positive.

• The satisfaction interview will be given one month after the baseline visit.

### Sample size

Two hundred seventy-six patients are required in each arm to detect a reduction of 10% in antibiotic prescription from 85% in the control group to 75% in the intervention group, assuming a rate of 85% of prescription in the control group, allowing 10% for losses in the follow-up visits, with a power of 90% and a level of significance of 5% (2-sided) and performance values of the RADT given by the manufacturer.

### Randomization

The unit of randomization will be the primary health care center, which will be allocated to the control or intervention group by a random sequence generated by a computer program. This unit of randomization will be taken into account in order to minimize contamination between these two groups. It may not be appropriate to randomize individuals since patients who attend the same primary health care center may be treated in the same way. Patients who meet the inclusion criteria will be consecutively selected by the participating physicians and allocated to the treatment group corresponding to the center.

### Blinding

It is not possible to blind participants, patients or doctors. Data will be analyzed blinded to treatment group allocation.

### Withdrawals

Patients will be free to withdraw from the study at any time. For obtaining the patient satisfaction data the patients will be phoned at least five times in three different timetables before being considered as withdrawal.

### Analysis

Data will be analyzed in accordance with CONSORT guidelines. The primary end point for the trial will be at baseline and at three weeks (prescription or not of antibiotic, RADT use and result, and culture result). Descriptive statistics of the outcome measures will be performed as well as baseline characteristics and clinic measures, calculating means and standard deviations or percentages, with 95% confidence intervals at each assessment for all the patients and for each group. Chi-square tests will be carried out to assess the impact of RADT on the percentage of antibiotic prescription in the intervention and control arms. For assessing the cost-effectiveness study of RADT direct costs such as antibiotics prescribed, RADT costs, and number of follow-up visit and indirect costs such as sick leave days will be taken into account. The different antibiotics prescribed, the GABHS resistance rates, the clinical recurrences and the clinical criteria will also be determined to assess the secondary objectives. The level of significance of all models will be set at 5%. Statistical analysis will be carried out with SPSS v. 15 (SPSS Inc., Chicago, IL) and the SAS v. 9.1.3 for Windows (SAS Institute Inc., Cary, NC, USA).

### Risks

We do not believe that the participants will be exposed to excessive risk by participation in this study since the visit schedule dictated by the protocol is similar to that undertaken in daily clinical practice. Neither do we foresee any risk to the patients in obtaining the pharyngeal sample.

### Study limitations

Acute pharyngitis is a very common process and we will be able to obtain the sample size required in this protocol without difficulty. In addition, the protocol is adapted to the way in which primary care physicians work and therefore implies neither ethical nor legal problems for its undertaking in our setting. The only difference with the usual practice will be the performance of RADT and the collection of the sample for culture. However, both procedures are relatively rapid and simple and are widely used in other countries. We hope there will not be any logistic problems with the submission of samples from the health care centers to the laboratory for culture since we will be personally responsible for this task. There are no major differences in the GABHS detection technique in the different laboratories of our setting and thus, no bias in this sense is expected. Centers and practitioners willing to participate may be more motivated professionals and will more likely to follow current guidelines and follow better clinical practice. This aspect could reduce the impact of the results obtained in relation to the optimization of treatment of acute pharyngitis with RADT. We do not expect many dropouts since the protocol is very similar to usual practice.

### Ethical and organizational review

The study will be conducted according to guidelines of the Helsinki Declaration and the Good Clinical Research Practice. The Project has been approved by the Ethical and Clinical Research Committee of the Jordi Gol Institute of Research in Primary Health Care. The certificate number is P06/03.

Informed consent: The information will be provided orally as well as written. Study subjects will have sufficient opportunity to ask questions regarding study details. Informed consent follows the guidelines of Helsinki Declaration and the rules of Title I, Article 12 of the Spanish Royal Decree 561 of the 16th April 1993

Data confidentiality: Individual data will be coded to ensure anonymity. Only researchers and monitors will have access to the data.

## Discussion

This article presents a detailed description of a randomized clinical trial designed to explore the impact of RADT on antibiotic prescribing in acute pharyngitis in adults. The trial proposed is sufficiently powered and, to the best of our knowledge, the first to allow conclusions on the effect of RADTs in adults with pharyngitis. Pharyngotonsillitis is a very prevalent disease and is one of the most frequent causes of medical visits in primary care. Many patients in our country visit the physician in search of antibiotic prescription since they believe that this is the treatment of choice, particularly among patients with multiple disease recurrence. In other cases, patients seek home remedies or go directly to the pharmacy to obtain medications to alleviate the sore throat that accompanies pharyngitis. Primary care physicians are aware that when the patients visit they wish to receive effective treatment with medications which rapidly alleviate the accompanying symptomatology and, in many cases, what they want is treatment with an antibiotic. This is probably the main reason why physicians in our country mainly prescribe these medications together with the fact that it is clinically difficult to distinguish streptococcal from viral etiology. We believe that this clinical trial will be of great use to know whether the RADT are truly effective in reducing the percentage of antibiotic prescription in our setting.

## Abbreviations

(RADT): Rapid antigen detection test; (GABHS): Group A beta-hemolyticstreptococcus.

## Competing interests

The Rapid Test Device OSOM StrepA of Genzyme will be kindly provided by Leti.

## Authors' contributions

JC and CL had the original idea of the study, obtained funding from the University and Innovation Department and from the Catalan Society of Family Medicine and helped with the drafting of the protocol. MB, JM and MG obtained funding from the University and Innovation Department and from the Catalan Society of Family Medicine and helped with the drafting the protocol. JM is main responsible as well as coordinator study group, CL helped with the drafting of the protocol. All authors have read and approved the final manuscript.

## Pre-publication history

The pre-publication history for this paper can be accessed here:

http://www.biomedcentral.com/1471-2296/11/25/prepub
